# Multi-Channel Cellytics for Rapid and Cost-Effective Monitoring of Leukocyte Activation

**DOI:** 10.3390/bios15030143

**Published:** 2025-02-24

**Authors:** Hojin Cheon, Samir Kumar, Inha Lee, Sanghoon Shin, Hyeji Jang, Young-Sun Lee, Myung-Hyun Nam, Hyun Sik Jun, Sungkyu Seo

**Affiliations:** 1 Department of Electronics and Information Engineering, Korea University, Sejong 30019, Republic of Korea; cheon_hj@korea.ac.kr (H.C.); skumar@korea.ac.kr (S.K.); ghost10s@korea.ac.kr (S.S.); hyyyeji0214@korea.ac.kr (H.J.); 2 Department of Biotechnology and Bioinformatics, Korea University, Sejong 30019, Republic of Korea; dlsgk1017@korea.ac.kr; 3 Department of Gastroenterology and Hepatology, Guro Hospital, Korea University College of Medicine, Seoul 08308, Republic of Korea; lys810@korea.ac.kr; 4 Department of Laboratory Medicine, Anam Hospital, Korea University College of Medicine, Seoul 02841, Republic of Korea; yuret@korea.ac.kr

**Keywords:** lens-free shadow imaging technology, leukocyte activation, multi-channel Cellytics, shadow parameters, cell morphology

## Abstract

Morphological changes in leukocytes are valuable markers for diseases and immune responses. In our earlier work, we presented Cellytics, a device that uses lens-free shadow imaging technology (LSIT) to monitor natural killer cell activity. Here, we present an improved Cellytics system that has been upgraded to a four-channel configuration to achieve higher throughput while maintaining robust reproducibility for rapid and cost-effective leukocyte analysis. The performance of this multi-channel Cellytics system was improved through refinements to the micro-pinhole chip. Etched pinholes provided better image resolution and clarity compared to drilled pinholes. To stimulate leukocytes, we used an activation stimulator cocktail (ASC) and quantified the resulting morphological changes using shadow-based metrics, including peak-to-peak distance (PPD) and maxima-to-minima standard deviation (MMD-SD). In addition, we developed a new leukocyte activation parameter (LAP) to specifically assess these activation-induced morphological changes. After ASC stimulation, leukocytes showed significantly increased PPD and LAP values and decreased MMD-SD compared to non-activated leukocytes. These results are consistent with the results of the flow cytometric analysis. These results emphasize the potential of Cellytics for the rapid and accurate assessment of leukocyte activation and provide a valuable tool for both clinical diagnostics and basic immunological research.

## 1. Introduction

Changes in cell morphology are important indicators of various diseases and serve as valuable tools for diagnosis and prognosis [1]. Morphological analysis offers crucial insights into the cellular functional state, enables predictions about disease progression, and offers information about underlying genetic, environmental, or pathological conditions [2,3,4,5]. Advanced techniques for morphological analysis are essential in providing diagnostic and prognostic information for a range of diseases, including malignancies, metabolic disorders such as diabetes, and inflammatory processes [6,7,8,9].

The recent COVID-19 pandemic has increased the focus on understanding the long-term impact of the virus on organ systems and emphasized the urgent need for innovative morphological analysis techniques to assess cellular damage and recovery [10,11,12]. Research has found remarkable numerical and morphological changes in leukocytes in individuals affected by COVID-19, with differences between mild and severe cases [13,14,15]. Therefore, a daily complete blood count (CBC) with manual leukocyte differentiation is recommended for hospitalized COVID-19 patients to monitor changes that may predict adverse outcomes and disease progression [16,17]. Recent studies also highlight leukocyte morphology and related parameters as potential indicators of disease severity and progression [18,19].

In clinical practice, leukocyte morphology is primarily assessed through CBCs with differential counting, with microscopic examination to identify the cell types and their characteristics [20]. This method provides valuable information about a patient’s general health and helps detect abnormalities indicative of infections or hematologic disorders. Therefore, monitoring leukocyte morphology from the blood count is crucial to making treatment decisions and improving the diagnosis of leukocyte disorders, as abnormal morphology may indicate underlying problems and infections. However, conventional methods of assessing leukocyte morphology cannot detect subtle cellular changes. This limitation emphasizes the need for advanced imaging techniques and machine learning algorithms to provide more detailed insights into cellular changes [21,22,23]. Furthermore, conventional microscopy is limited in throughput, resolution, and field of view (FOV), which hinders the efficient analysis of large cell populations [24,25]. Manual microscopy is also labor-intensive, subjective, and requires highly skilled personnel; the inherent variability in manual assessments can affect accuracy. While manual microscopy is still a valuable tool, it is being supplemented by automated and digital systems that improve efficiency and accuracy, especially in high-throughput environments, by reducing labor and speeding turnaround times for diagnosis [26,27,28].

Lens-free technology (LSIT) is emerging as a cost-effective, portable, and efficient approach to imaging and analyzing cells [29,30,31,32,33]. LSIT is particularly advantageous for medical diagnostics [34], cell monitoring [35], and telemedicine applications [36], as it eliminates the need for complex devices or staining reagents [37]. LSIT uses computer-assisted techniques to capture and analyze cell images without conventional lenses, reducing costs and simplifying imaging [38,39].

LSIT is a variant of digital inline holography (DIH), a lens-free imaging method that records interference patterns of light scattered by microscopic objects, termed shadow patterns [40,41]. By eliminating lenses, DIH reduces the size and cost of the system, making compact and portable devices possible. It captures phase and amplitude information, facilitating 3D imaging and quantitative analysis of biological samples, including cell size and shape [42]. This lens-free approach significantly reduces system costs and provides a larger FOV than conventional microscopes, enabling high-throughput analysis of large numbers of cells. LSIT extracts valuable information about the size, shape, and other properties of cells from these shadow images, supporting various applications in cell analysis [43].

In previous research, we successfully developed and used Cellytics, an LSIT-based device, to identify NK and CD34+ cells and continuously monitor cells [44,45]. However, as it is a single-channel device with only one analysis path, the throughput for the simultaneous processing of several samples was limited. To overcome these limitations, we are introducing a multi-channel version of Cellytics that expands analytical capabilities and enables simultaneous analysis, increasing throughput.

This study has two main aims: First, to evaluate the performance of multi-channel Cellytics by examining parameters such as image sharpness and the ability to identify and analyze microspheres. Secondly, we aim to investigate the potential of this improved device for direct analysis of leukocyte morphology, particularly in the context of leukocyte activation, using an activation stimulator cocktail (ASC). We investigate LSIT-derived shadow parameters, including peak-to-peak distance (PPD) and maxima-to-minima standard deviation (MMD-SD), to distinguish between activated and non-activated leukocytes. In addition, we introduce a new shadow parameter, the leukocyte activation parameter (LAP), to quantify leukocyte activation in response to ASCs. This work aims to advance LSIT-based blood cell analysis and provide a technical basis for rapid and cost-effective leukocyte analysis.

## 2. Materials and Methods

### 2.1. Cell Preparation and Activation

#### 2.1.1. Blood Sample Collection and Leukocyte Isolation

Whole blood samples were obtained from healthy donors in collaboration with Korea University Anam Hospital (Institutional Review Board approval number: 2021AN0040). Leukocytes were isolated from whole blood by erythrocyte lysis using RBC Lysis Buffer (BioLegend, San Diego, CA, USA). The whole blood was diluted 1:20 in RBC Lysis Buffer, mixed gently, and incubated for 12 min at room temperature. Samples were centrifuged at 300× *g* for 5 min at 4 °C to separate erythrocytes and platelets. The resulting leukocyte pellet was washed twice with phosphate-buffered saline to ensure that the residual erythrocytes and platelets were completely removed, resulting in a purified leukocyte sample.

#### 2.1.2. Activation Stimulator Cocktail (ASC) Preparation

The ASC (Metaimmunetech, Sejong, Republic of Korea) was formulated based on compositions previously developed for NK cell studies. It comprised recombinant human IL-2 (200 U/mL; Peprotech, Inc., Cranbury, NJ, USA), recombinant human IL-12 (2 ng/mL; R&D Systems, Inc., Minneapolis, MN, USA), PMA (50 ng/mL; Sigma-Aldrich, St. Louis, MO, USA), and ionomycin (500 ng/mL; Sigma-Aldrich, St. Louis, MO, USA). ASC was used in four concentrations: 100%, 75%, 50%, and 25% [44]. For the 100% concentration, 10 μL of ASC was added to 1 mL of the leukocyte sample. For the 50% concentration, a mixture of 5 μL ASC and 5 μL cell culture medium was added to 1 mL of the sample.

#### 2.1.3. Leukocyte Activation Procedure

**Figure 1:**
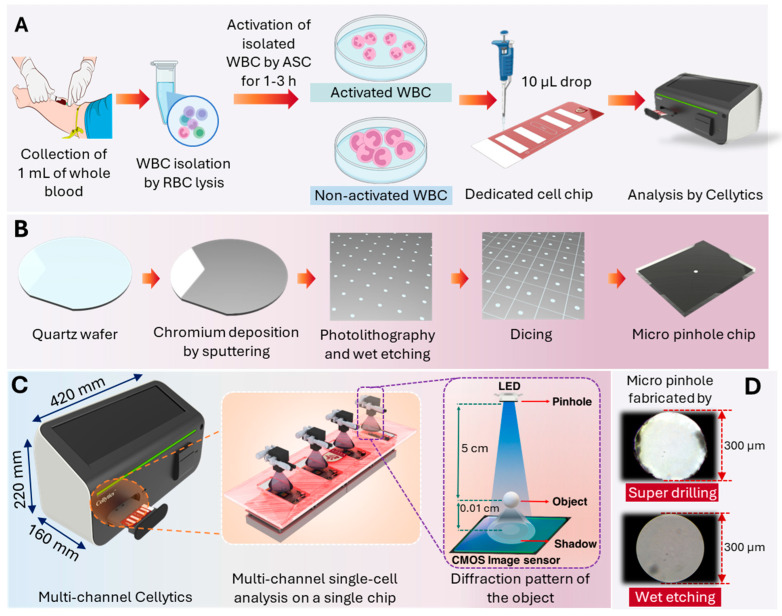
Multi-channel Cellytics device and experimental workflow. (**A**) Schematic representation of the experimental workflow for Cellytics analysis of leukocytes, including leukocyte isolation, ASC activation, loading onto the dedicated cell chip, and Cellytics analysis. (**B**) Step-by-step illustration of micro-pinhole chip fabrication by wet etching on a quartz wafer, with detailed chromium deposition by sputtering and wet etching. (**C**) Schematic of the multi-channel Cellytics device showing overall dimensions, key components, and a diagram illustrating diffraction pattern formation. (**D**) Comparative microscopy images of 300 µm micro-pinholes made by super drilling and wet etching.

Isolated leukocytes were activated using ASC. Since neutrophils comprise approximately 50–70% of the total leukocyte population, the ASC components (IL-2, IL-12, PMA, and ionomycin) were selected to stimulate a broad range of leukocyte subtypes, including neutrophils (directly via PMA and ionomycin and indirectly via IL-2 and IL-12 through T cells, Th1 cells, and NK cells). This strategy allowed for the assessment of overall leukocyte activation and provided insights into ASC-mediated neutrophil activation within a heterogeneous leukocyte population. The experimental workflow for the Cellytics analysis of leukocyte isolation, ASC activation, loading onto a dedicated cell chip, and subsequent Cellytics analysis is shown in Figure 1A.

#### 2.1.4. Shadow Parameter Selection and Comparative Analysis

Upon activation, neutrophils undergo characteristic morphological changes, including an increase in cell size (due to enlargement of the cytoplasm and reorganization of the cytoskeleton) and a decrease in internal complexity (due to degranulation of the granules). These changes show up in flow cytometry as increased forward scatter (FSC) and decreased side scatter (SSC). In this study, Cellytics was used to analyze the corresponding shadow parameters: PPD and MMD-SD. The PPD, which correlates linearly with cell size, was determined by analyzing the pixel intensity difference between the central maximum and minimum of the diffraction pattern. The MMD, defined as the maxima-to-minima distance, evaluates the internal complexity by quantifying the difference between the minimum and maximum values of the first-order diffraction patterns. The MMD-SD was used to assess cell shape; lower MMD-SD values indicate a more circular morphology, while higher values indicate a more elongated shape.

#### 2.1.5. Statistical Analysis

All statistical analyses were performed using Prism 8 software (GraphPad Software, San Diego, CA, USA). Group differences were assessed using the non-parametric Mann–Whitney U test. Statistical significance was defined as *p* < 0.05. For the measurements of cellular parameters (PPD, MMD-SD, and LAP), at least 500–1000 individual cells were analyzed, and the SD calculated. Flow cytometry data were processed using FlowJo software (v10.0) to calculate mean values and SD.

### 2.2. Multi-Channel Cellytics Enhancement

#### 2.2.1. Micro-Pinhole Chip Fabrication

Micro-pinhole chips for the optical four-channel system were fabricated on 0.5 mm thick quartz disks using a wet etching process. Figure 1B outlines the wet etching fabrication process. A 100 nm thick chromium layer was sputtered onto the quartz wafer, followed by photoresist coating and UV exposure through a photomask. The development of the photoresist defined the hole pattern. Quartz was chosen as the substrate material due to its optical transparency and chemical inertness. Chromium was selected as the mask material because it is very resistant to etching and can be easily deposited [46]. The exposed chrome was then selectively removed by wet etching, resulting in uniform pinholes with a diameter of 300 μm. After removing the remaining photoresist and cleaning the wafer, the patterned and etched wafer was cut into 5.8 mm × 6.8 mm chips. The final design of the pinhole chip, as shown in Figure 1B, ensures optical compatibility with the LED and CMOS image sensor in the Cellytics system. Commercially available pinholes did not have the required combination of size and material, so custom fabrication was required. In-house pinhole manufacturing also enabled the production of multiple pinholes on a single quartz wafer, increasing cost efficiency and facilitating integration into the multi-channel system.

#### 2.2.2. Pinhole Diameter Optimization

To optimize the pinhole diameter, a comparative analysis was performed using diameters from 100 μm to 400 μm in 50 μm increments. A specially developed circuit board with an integrated LED was used for this test setup. The micro-pinhole chips were imaged using an optical microscope at 400× magnification (CKX31SF, Olympus Co., Ltd., Tokyo, Japan).

#### 2.2.3. Comparison with Conventional Pinhole Design

Conventional pinholes, which are made of plastic by injection molding, are designed for integration into PCBs equipped with LEDs, resulting in a larger form factor. Drilling after injection molding leads to uneven cut surfaces. Microscopic examination at 200× magnification revealed that these conventional pinholes have irregular openings characterized by debris and irregularities (Figure 1D). Visual inspection confirmed remarkable differences in the quality and shape of the openings. Wet etching revealed pinholes with smoother edges, a more circular shape, and less debris than drilled pinholes. These differences in pinhole morphology can significantly affect the optical performance, with the improved circularity of wet-etched pinholes likely to produce sharper and more predictable diffraction patterns.

#### 2.2.4. Multi-Channel Cellytics System Description

Figure 1C shows a schematic representation of the multi-channel Cellytics device, with the dimensions and components described in detail. The multi-channel Cellytics system comprises four identical optical channels. Each channel is equipped with a 470 nm LED (LB W5SM-FZHX-35, Osram, Munich, Germany), a 300 μm pinhole aperture, and a monochrome 5-megapixel CMOS image sensor (MT9P031I12STM−DP, ONSEMI, Phoenix, AZ, USA). The LED, mounted on a customized circuit board and precisely aligned with the pinhole, converts the emitted incoherent light into semi-coherent illumination. This semi-coherent light illuminates the samples (10 μL per chamber) in a specially designed four-chamber chip mounted on a PCB close to the CMOS image sensor. The light passing through the pinhole causes the sample to project shadow diffraction patterns onto the sensor. The multi-channel Cellytics system captures shadow images of up to 1000 cells simultaneously within 20 s using proprietary software integrated into a built-in PC. This software processes the shadow images and quantifies the size and morphology of individual cells, enabling high-throughput cell analysis. Cellytics completes the analysis of each sample—from sample loading to data acquisition—in less than 5 min, whereas conventional flow cytometry typically requires 35 min per sample (Table S1) [47].

## 3. Results and Discussion

### 3.1. Pinhole Diameter Optimization

**Figure 2:**
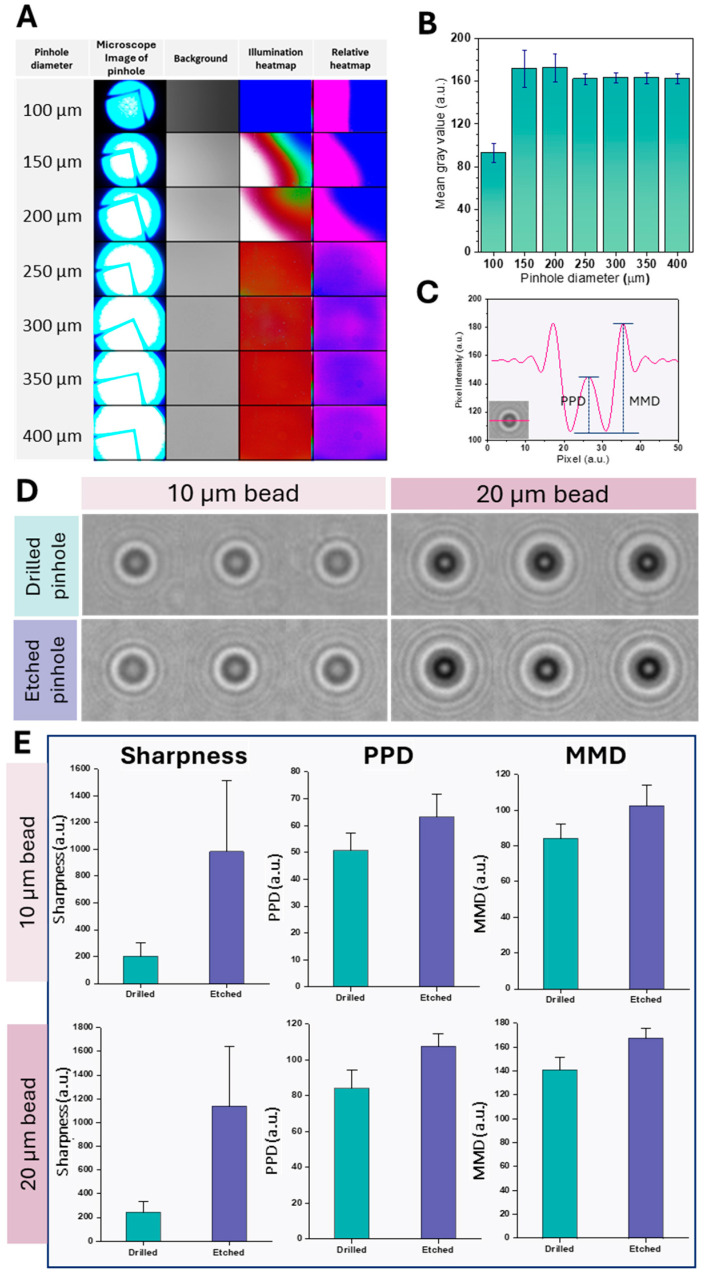
Pinhole characterization and optical performance assessment. (**A**) Characterization of pinholes with different diameters: representative microscope images, background illumination patterns, and corresponding heat maps depicting absolute and relative light intensity. (**B**) Plot showing the mean pixel gray value and standard deviation (SD) as a function of pinhole diameter and quantifying light transmission. (**C**) Illustration defining the peak-to-peak distance (PPD) and the maxima-to-minima distance (MMD) derived from a diffraction pattern. PPD stands for the distance between the two central intensity peaks, and MMD denotes the distance between the central maximum and a neighboring minimum. (**D**) Shadow images of 10 and 20 µm polystyrene beads taken using both drilled and etched pinholes to assess image quality. (**E**) Comparative analysis of sharpness, PPD and MMD values for drilled and etched pinholes using 10 µm and 20 µm beads as test samples. The “L”-shaped structure visible in the images represents the LED electrode. The data are given as mean ± SD.

Figure 2A shows microscopic images of the fabricated pinholes, background illumination patterns, and corresponding heat maps. A concomitant increase in light transmittance was observed with increasing pinhole diameter, reflected in brighter illumination heatmaps, a predictable result as larger apertures inherently transmit more light. Relative heat maps showed a more uniform light distribution for pinhole diameters of 300 µm and larger. These differences in light transmission and uniformity are critical in lens-free imaging applications, where consistent illumination is essential for precise particle sizing [47].

The relationship between pinhole diameter and mean gray value was further investigated (Figure 2B). A significant increase in mean gray value occurred between 100 µm and 150 µm pinhole diameter. This increase is due to increased diffraction effects at smaller diameters, resulting in lower gray values. While smaller pinholes can provide better resolution by minimizing blurring, they also reduce the signal-to-noise ratio (SNR) due to lower photon capture, which can reduce the mean gray value by amplifying image noise [48]. Conversely, larger pinhole diameters improve light and photon capture and, therefore, SNR, but can compromise resolution by increasing blur. This trade-off can produce a more uniform mean gray value as the noise is averaged over a larger area [49]. Beyond 150 µm, a further increase in the pinhole diameter up to 400 µm did not significantly change the mean gray value. This plateau indicates that increasing the pinhole diameter beyond 150 µm does not significantly improve signal intensity. Within the 150 µm to 400 µm range, the relatively stable mean gray value likely reflects an optimal balance where resolution remains adequate for image quality without excessive noise. Based on these results, a pinhole diameter of 300 µm was optimal for achieving a balance between signal intensity and illumination uniformity.

Figure 2D shows diffraction patterns of 10 µm and 20 µm polystyrene beads imaged using drilled and etched pinholes. Images with etched pinholes show sharper and better-defined diffraction rings than those obtained with drilled pinholes for both bead sizes, indicating better optical performance. This improvement is due to the greater uniformity and smoothness of the etched pinholes compared to the drilled pinholes. This uniformity minimizes edge roughness, a critical factor in reducing wavefront errors and improving the quality of diffracted light. Edge roughness in pinholes can cause aberrations such as trefoil and coma, which affect image quality [48]. The smoother edges of etched pinholes also reduce light scattering, which is crucial for achieving sharper images with smaller central spot sizes. The scattering from irregular edges of drilled pinholes can result in a broader and less defined point spread function, negatively impacting spatial resolution [49]. The improved image quality achieved with etched pinholes is essential for clearly defining shadow parameter boundaries.

Figure 2E quantifies sharpness, PPD, and MMD values for drilled and etched pinholes using 10 µm and 20 µm beads. Etched pinholes consistently exhibited higher sharpness, PPD, and MMD values than drilled pinholes for both bead sizes. As previously mentioned, the higher sharpness, PPD, and MMD values of etched pinholes indicate improved image resolution and contrast, likely due to smoother and more uniform apertures. These quantitative results confirm the qualitative observations from Figure 2D, which showed sharper diffraction images with etched pinholes. To evaluate inter-channel reproducibility, shadow images of 20 µm polystyrene beads were acquired in all four channels (*n* = 3), and PPD, MMD-SD, and LAP values were calculated. Minimal deviations were observed with a coefficient of variation (CoV) of less than 10%, confirming the consistency of the measurements across the channels (Figure S1).

### 3.2. Leukocyte Activation Assessment Using LSIT-Derived Shadow Parameters

**Figure 3:**
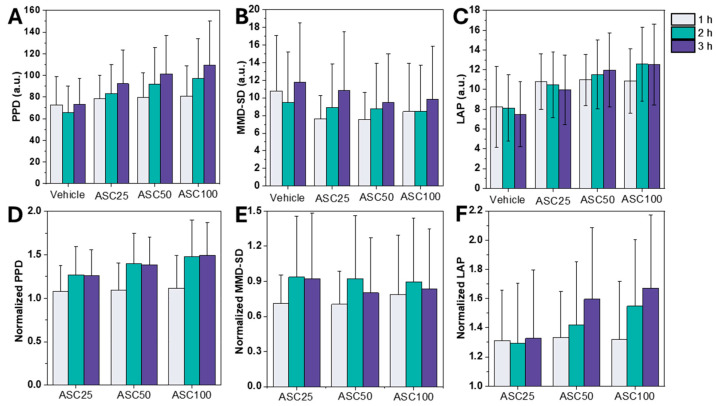
Effect of ASC stimulation on leukocyte shadow parameters. (**A**–**C**) Diagrams showing PPD, MMD-SD, and LAP values for leukocytes stimulated with different ASC concentrations (25%, 50%, and 100%) and incubation times (1, 2, and 3 h) to evaluate activation kinetics. (**D**–**F**) Plots of normalized PPD, MMD-SD, and LAP values allow for a direct comparison of relative changes induced by ASC. The data are given as mean ± SD.

Figure 3 compares leukocyte activation as measured by LSIT-derived shadow parameters. PPD and MMD-SD, key parameters, effectively distinguish between activated and non-activated leukocytes. Figure 3A shows the PPD values at different ASC stimulation periods. There was a statistically significant increase in PPD after one hour of ASC treatment, suggesting that PPD is a sensitive indicator of leukocyte activation that reflects ASC-induced cellular morphologic changes. PPD has a linear relationship with the actual size of micro-objects and is thus a reliable measure of cell size [36]. PPD values increased with increasing ASC concentration, peaking at ASC100 (Figure 3A), demonstrating a time-dependent effect of ASC on leukocyte morphology.

In contrast to the PPD trends, MMD-SD initially decreased within the first hour after stimulation and subsequently stabilized, highlighting its potential for detecting structural dynamics during activation (see Figure 3B). MMD-SD, indicative of internal complexity, showed a less pronounced trend and a slight decrease with increasing ASC concentration. This decrease could correlate with the increased cell size observed in ASC-stimulated samples, which could reduce the apparent internal complexity compared to non-stimulated controls. The stable or decreasing trend in MMD-SD suggests that ASC stimulation does not significantly alter the variability of maxima-to-minima distances in the light scatter patterns, indicating that ASC primarily affects other aspects of leukocyte morphology or internal structure beyond the complexity captured by MMD-SD.

Although conventional shadow parameters such as PPD and MMD-SD describe certain aspects of leukocyte activation, they cannot fully capture the morphological changes. Therefore, LAP was developed as a composite metric that integrates these shadow parameters to comprehensively assess both changes in cell size and internal complexity following leukocyte activation. LAP values increased with ASC concentration, mirroring the trend observed for PPD. In addition, the effect of incubation time was more pronounced for LAP, with a marked increase observed between one and two hours at higher ASC concentrations (Figure 3C). This increased sensitivity of LAP to incubation time was compared to PPD, and MMD-SD suggests that LAP is more responsive to time-dependent changes in leukocyte activation.

Normalization of the data facilitated a comparison of the relative changes induced by ASC and showed a comparable increase in PPD and LAP. Normalized PPD and LAP showed a clear upward trend with increasing ASC concentration, while normalized MMD-SD showed a slight decrease or remained stable (Figure 3D–F). Overall, these shifts in the shadow parameters (PPD, MMD-SD, and LAP) serve as robust indicators of leukocyte activation. The observed increases in PPD and LAP reflect changes in cell size and internal complexity and are consistent with previous studies showing that leukocyte activation is accompanied by significant changes in cell morphology [44,48].

These results highlight the potential usefulness of LSIT shadow parameters in clinical diagnostics to differentiate between activated and non-activated leukocytes. While PPD and MMD-SD each capture different morphological changes, LAP provides a consolidated measure of leukocyte activation and minimizes the variability associated with the measurement of individual parameters. Given its strong correlation with activation status, LAP emerges as a promising diagnostic biomarker for immune responses, enabling more precise leukocyte activation monitoring in clinical and research settings. Although these results provide valuable insights into the effects of ASC on leukocyte shadowing parameters, future research should investigate the underlying cellular mechanisms driving these observed changes and explore the influence of different stimuli.

### 3.3. Comparison Between Flow Cytometry and PPD vs. MMD-SD Density Plots

**Figure 4:**
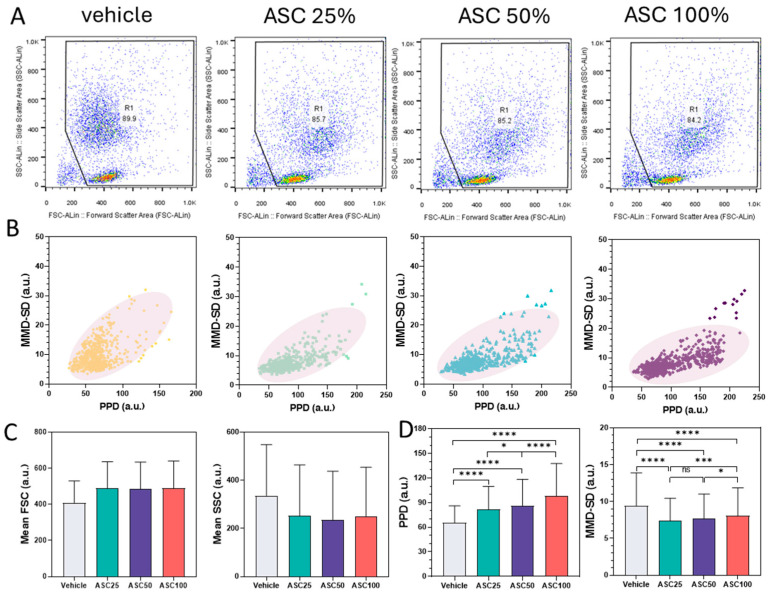
Correlation analysis of FACS and LSIT-derived shadow parameter data for leukocyte activation. (**A**) Flow cytometric dot plots (FSC-ALin vs. SSC-ALin) depicting leukocyte populations for vehicle control and increasing ASC concentrations (25%, 50%, and 100%). (**B**) Corresponding density plots (PPD vs. MMD-SD) derived from LSIT measurements showing population shifts upon ASC stimulation. (**C**) Bar graphs showing the mean forward scatter (FSC) and side scatter (SSC) values from FACS analysis at different ASC concentrations. (**D**) Bar graphs showing the mean PPD and MMD values from LSIT analysis for the corresponding ASC concentrations. Data are presented as mean ± SD; statistical significance was determined using the non-parametric Mann–Whitney U test. ns = not significant; * *p* < 0.05, ** *p* < 0.01, *** *p* < 0.001 and **** *p* < 0.0001.

Figure 4 compares data from leukocyte activation experiments, contrasting flow cytometry analyses (Figure 4A) with PPD vs. MMD-SD density plots (Figure 4B) and correlations between forward/side scatter and PPD/MMD-SD (Figure 4C,D) after two hours of ASC stimulation. Leukocytes were analyzed by fluorescence-activated cell sorting (FACS), with forward scatter (FSC) and side scatter (SSC) measured using the Guava^®^ EasyCyte™ system (Millipore, Burlington, MA, USA) and data processed in FlowJo (v10.0). These analyses compare the leukocyte populations before and after activation with ASC.

FSC generally correlates with cell size, while SSC reflects internal complexity or granularity [49,50]. Increasing ASC concentration resulted in increased FSC values, indicating larger cell size, and a slight decrease in SSC, indicating lower cellular complexity. The percentage of cells within the R1 gate decreased slightly after activation (e.g., from 89.9% to 84.2%), indicating increased heterogeneity or the emergence of subpopulations. This downward shift in scattering intensity is due to cell deformation, changes in granularity, or altered refractive properties because of activation, associated with changes in cell size or granularity. These changes are consistent with leukocyte activation, leading to altered physical properties that can be detected by flow cytometry.

In the PPD vs. MMD-SD density plots, the data points shifted to higher PPD and lower MMD-SD values with increasing ASC concentration, reflecting the trends in the FACS data (Figure 4B). The increase in PPD corresponds to the increase in FSC (cell size), and the decrease in MMD-SD corresponds to the decrease in SSC (cellular complexity/granularity). The evolving position and shape of the density ellipses, representing the highest data concentration areas, further illustrate these changes. Activated leukocytes exhibit more compact or displaced clusters compared to non-activated cells, indicating more uniform morphological changes upon activation. The tilt of the cluster toward the PPD axis signifies an increase in size (correlated with forward scatter), whereas the concomitant decrease in MMD-SD values reflects decreased internal complexity (correlated with side scatter). This density shift supports the hypothesis that ASC-induced activation causes measurable morphological changes. The downward population shift observed in the PPD vs. MMD-SD scatter plots is consistent with the FACS results.

Figure 4C,D show the mean FSC, SSC, PPD, and MMD-SD values for the vehicle control and three ASC concentrations (ASC25, ASC50, and ASC100). The trends in PPD and MMD-SD parallel the trends observed in mean FSC and mean SSC from the FACS analysis: a slight increase in mean FSC and PPD with increasing ASC concentration, coupled with a decrease in mean SSC and MMD-SD. This concordance supports the hypothesis that ASC stimulation leads to changes in cell morphology. The increase in FSC and PPD indicates an increase in cell size (due to cell swelling and an increase in cytoplasm). In contrast, the decrease in SSC and MMD-SD indicates decreased internal complexity (possibly due to reduced granularity). These results are consistent with the observations in Figure 4A,B.

The observed correlation between FACS data and PPD/MMD-SD suggests that ASC can induce changes in the cell membrane that lead to changes in cell size and complexity. Although the trends are similar, the magnitude of changes in PPD and MMD-SD may not be directly proportional to the changes in FSC and SSC due to inherent differences in measurement principles or sensitivity of the technique. It is critical to recognize that PPD and MMD-SD are indirect measures of cell size and complexity; although they correlate well with FSC and SSC, they may not capture all aspects of cell morphology assessed by flow cytometry.

### 3.4. Flow Cytometry Analysis of CD64 and CD66b Expression and Correlation with LAP

**Figure 5:**
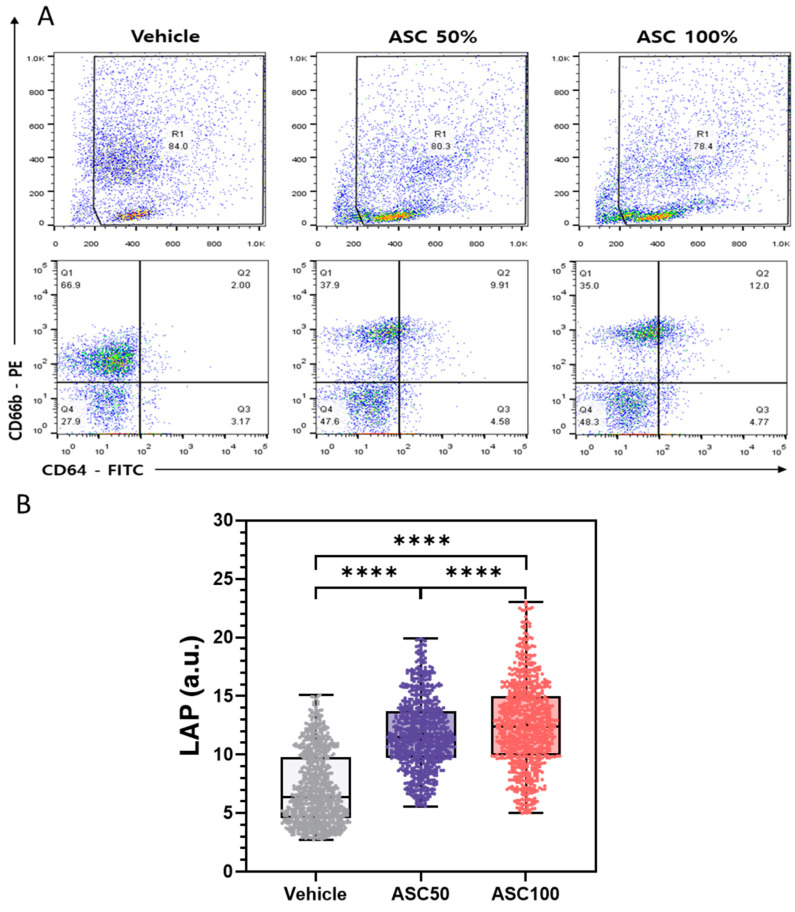
Comparative assessment of leukocyte activation using flow cytometry and LAP. (**A**) Flow cytometric analysis of CD64 and CD66b expression on leukocytes. Top panels: Scatter plots showing R1-gated leukocyte populations. Bottom panels: Quadrant analysis delineating CD64^+^/CD66b^−^ (monocytes/macrophages), CD64^+^/CD66b^+^ (activated neutrophils), CD64^−^/CD66b^−^ (double negative CD64/CD66b population, potentially lymphocytes), and CD64^−^/CD66b^+^ (resting granulocytes) cell populations under vehicle control, 50% ASC, and 100% ASC conditions. (**B**) Rain cloud plots showing the distribution of LAP values at the corresponding ASC concentrations. The boxes represent the interquartile range (IQR); the horizontal lines indicate the median; the whiskers extend to 1.5 times the IQR; the dots represent individual data points. Data are presented as mean ± SD; statistical significance was determined using the non-parametric Mann–Whitney U test. ns = not significant; * *p* < 0.05, ** *p* < 0.01, *** *p* < 0.001 and **** *p* < 0.0001.

Figure 5A shows the flow cytometric analysis of CD64 and CD66b expression on leukocytes under different ASC stimulation conditions after two hours of stimulation. The upper panels show scatter plots with the R1 gate delineating the leukocyte population analyzed, and the percentage of cells within R1. The bottom panels show a quadrant analysis based on CD64 and CD66b expression with the percentage of cells in each quadrant indicated (Q1: CD64^−^/CD66b^+^, Q2: CD64^+^/CD66b^+^, Q3: CD64^+^/CD66b^−^, Q4: CD64^−^/CD66b^−^).

With increasing ASC concentration, a significant increase in the CD64^+^/CD66b^+^ population (activated neutrophils) (Q2) was observed, accompanied by a decrease in the CD64^−^/CD66b^+^ population (resting granulocytes) (Q1). The CD64^+^/CD66b^−^ population (monocytes/macrophages) (Q3) remained relatively stable (3–5%), indicating minimal monocyte activation under these experimental conditions. The double negative (CD64^−^/CD66b^−^) population (Q4) increased from 28% to 48% upon ASC activation. This seemingly counterintuitive increase can be attributed to several possibilities, including downregulation (shedding, internalization, or altered gene expression) of CD64 or CD66b on activated cells, shifting them to the double negative quadrant [51,52]. Alternatively, a reduction in CD64^+^ or CD66b^+^ cell populations due to cell death (and subsequent marker loss) could also contribute to the relative increase in double-negative cells. These observed changes suggest that ASC stimulation may induce a distinct neutrophil subpopulation characterized by loss of CD64 and CD66b expression. Further studies are needed to fully describe this subpopulation and its functional role. The observed increase in Q2 and decrease in Q1 strongly suggest that ASC effectively triggers neutrophil activation and converts quiescent granulocytes to an activated state.

Figure 5B illustrates the distribution of LAP values across different ASC concentrations. The mean LAP value and data dispersion (indicated by IQR and data point distribution) increased with increasing ASC concentration, demonstrating a dose-dependent effect of ASC on leukocyte activation. These results are consistent with the FACS analysis (Figure 5A) and the analysis of PPD and MMD-SD (Figure 4), confirming the conclusion that ASC effectively activates leukocytes. The LAP parameter derived from shadow images using Cellytics serves as a reliable indicator of leukocyte activation and shows a strong correlation with established methods such as FACS. This indicates the potential of LAP as a valuable tool for quantifying leukocyte activation. The greater scatter of data at higher ASC concentrations could indicate a more heterogeneous response to ASC stimulation, possibly due to different sensitivity or activation states of individual cells. The use of shadow parameters derived from shadow images offers potential advantages over conventional methods such as FACS, including label-free analysis and a simplified experimental setup. The observed correlation between shifts in cell populations in FACS and changes in LSIT shadow parameters highlights the potential of LSIT to provide comparable information on leukocyte activation using a label-free approach. This could be particularly beneficial for high-throughput screening or in situations with limited antibody availability. While this study provides valuable insight into the effects of ASC on leukocyte activation, further research is needed to validate the clinical utility of LSIT and LAP in larger and more diverse patient populations, including those with different disease states.

## 4. Conclusions

This study introduces an LSIT-based multichannel device for the rapid and cost-effective analysis of leukocytes. By optimizing micro-pinhole chip parameters—particularly through the use of etched pinholes—we enhanced image quality and measurement accuracy. Compared to drilled pinholes, etched pinholes generate sharper diffraction patterns, resulting in more reliable data. A pinhole diameter of 300 μm was identified as optimal, striking a balance between light transmission and resolution. Furthermore, we established the shadow parameter LAP, which integrates PPD and MMD-SD, as a highly sensitive marker of leukocyte activation, demonstrating a clear dose-dependent response to ASC stimulation. The strong correlations between LSIT-derived parameters (PPD, MMD-SD, and LAP) and flow cytometric measurements (FSC and SSC) further validate the accuracy of our approach. Given its label-free detection and quantification capabilities, LSIT presents significant advantages for high-throughput screening and point-of-care diagnostics, making it a promising tool for clinical and research applications.

## Data Availability

Data will be made available upon request.

## Supplementary Materials

The following supporting information can be downloaded at: https://www.mdpi.com/article/10.3390/bios15030143/s1, Figure S1: Inter-channel Reproducibility of Multi-channel Cellytics Measurements; Table S1: Comparative Processing Time Analysis: Multi-channel Cellytics versus Flow Cytometry.
