# Supplementary material for: Multi-Channel Cellytics for Rapid and Cost-Effective Monitoring of Leukocyte Activation
Source: Biosensors (Basel). 2025 Feb 24;15(3):143. doi: 10.3390/bios15030143 (PMC11940678; doi:10.3390/bios15030143)
Supplement: Supplementary file 1 [file biosensors-15-00143-s001.zip › biosensors-3428289-supplementary.pdf]

Supplementary information for

Multi-Channel Cellytics for Rapid and Cost-Effective Monitoring of Leukocyte Activation

Hojin Cheon [1,†], Samir Kumar [1,†], Inha Lee [2], Sanghoon Shin [1], Hyeji Jang [1], Young-Sun Lee [3], Myung-Hyun Nam [4], Hyun Sik Jun [2,*] and Sungkyu Seo [1,*]

[1] Department of Electronics and Information Engineering, Korea University, Sejong 30019, Republic of Korea; cheon_hj@korea.ac.kr (H.C.); skumar@korea.ac.kr (S.K.); ghost10s@korea.ac.kr (S.S.); hyyyeji0214@korea.ac.kr (H.J.)
[2] Department of Biotechnology and Bioinformatics, Korea University, Sejong 30019, Republic of Korea; dlsgk1017@korea.ac.kr
[3] Department of Gastroenterology and Hepatology, Guro Hospital, Korea University College of Medicine, Seoul 08308, Republic of Korea; lys810@korea.ac.kr
[4] Department of Laboratory Medicine, Anam Hospital, Korea University College of Medicine, Seoul 02841, Republic of Korea; yuret@korea.ac.kr
* Correspondence: toddjun@korea.ac.kr (H.S.J.); sseo@korea.ac.kr (S.S.); Tel.: +82-44-860-1427 (H.S.J.); +82-44-860-1411 (S.S.); Fax: +82-44-860-1585 (H.S.J.); +82-44-860-1598 (S.S.)
† These authors contributed equally to this work.

Table S1. Comparative Processing Time Analysis: Multi-channel Cellytics versus Flow Cytometry.

**Method**	**Sample preparation (min)**	**Data acquisition (min)**	**Analysis (min)**	**Total Time (min)**
Flow cytometry	30–40	10–15	20–30	60–85
Multi-channel Cellytics	1–5	< 2	5–10	8–15

This table contains a comparative analysis of the processing time for leukocyte analysis using multi-channel Cellytics and conventional flow cytometry. Cellytics shows a significant reduction in overall processing time, primarily due to its label-free methodology and direct morphological analysis, which eliminates the need for antibody staining and extensive data processing. Overall analysis time is reduced by approximately 80–90% with Cellytics, highlighting the significant advantage of LSIT for rapid assessment of leukocyte activation.

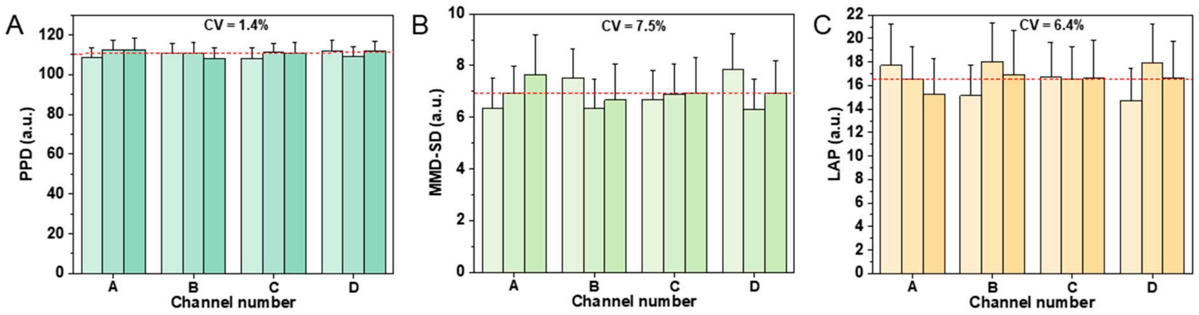

Figure S1. Inter-channel Reproducibility of Multi-channel Cellytics Measurements. (a) Peak-to-peak distance (PPD), (b) maxima-to-minima distance standard deviation (MMD-SD), and (c) leukocyte activation parameter (LAP) are plotted as a function of channel number (n = 3) to assess consistency between channels. The plots show minimal variation between channels for PPD, MMD-SD and LAP values with a coefficient of variation (CoV) consistently below 10%. This low CoV confirms the high reproducibility and consistency of the measurements across the four independent optical channels of the multi-channel Cellytics multichannel device.
